# A Case of Distal Epithelioid Sarcoma of the Thumb Expressing Podoplanin, TLE1 and Ca 125

**DOI:** 10.1155/2013/312786

**Published:** 2013-04-18

**Authors:** George Karagkounis, Theodore Argyrakos, G. Charkiolakis, O. Castana, D. Rontogianni

**Affiliations:** ^1^Department of Pathology, Evaggelismos Hospital, Ipsilantoy 45-47, 106 76 Athens, Greece; ^2^Department of Plastic Surgery, Evaggelismos Hospital, Ipsilantoy 45-47, 106 76 Athens, Greece

## Abstract

Distal epithelioid sarcoma is a rare and slowly growing tumor that usually develops in the upper extremities of young adults. Neoplastic cells have both spindle and epithelioid appearance and are characterized by the loss of the nuclear protein SMARCB1/INI1. We present the case of a distal epithelioid sarcoma arising in the thumb of a 14-year-old girl, which immunohistochemically was characterized by the loss of SMARCB1/INI1 protein as well as the expression of podoplanin (D2-40), TLE1, Glut1, and Ca 125; plus, we highlight the differential diagnosis of epithelioid sarcoma from its histological mimics.

## 1. Introduction 

Epithelioid sarcoma (ES) is a rare slowly growing soft tissue tumor that was first described by Enzinger in 1970 [1]. The conventional or distal form of ES typically appears as one or multiple painless nodules in the dermis or subcutis of the upper extremities, especially the hand and the wrist, of young adults [2]. An aggressive subtype of ES known as proximal/axial type arising in the soft tissues of pelvis, perineum, and proximal extremities of middle-aged patients was identified in 1997 [3, 4]. The cells of distal type ES are of spindle/polygonal morphology while those of the proximal type ES more often present with rhabdoid features. A possible link of the proximal type ES with rhabdoid tumors has been hypothesized [5] while several authors have demonstrated that proximal ES is a distinct entity [6]. ES presents a diagnostic challenge for both the clinician, who usually diagnoses such lesions as “indurated ulcers” or “infected warts,” as well as the pathologist, who delays to approach the correct diagnosis because of the epitheliod or necrotic granuloma-like appearance of the tumor [1, 5].

*SMARCB1/INI1* gene located on chromosome 22 is a tumor suppressor gene. Its biallelic inactivation is involved in the development of atypical teratoid tumors of the central nervous system and malignant rhabdoid tumors of renal or extra renal origin [7]. The protein of the gene *SMARCB1/INI1* constitutes an invariant subunit of the chromatin remodeling complexes [8], and its subsequent loss from the nucleus of the neoplastic cells can be immunohistochemically detected. Immunohistochemical loss of the SMARCB1/INI1 protein expression has been described in myoepithelial carcinomas and renal medullary carcinomas, in a subset of malignant peripheral nerve sheath tumors and of extraskeletal myxoid chondrosarcomas as well as in ES of both distal and proximal type. Reduced or less often loss of nuclear expression has also been described in synovial sarcomas [9, 10].

## 2. Case Presentation

### 2.1. Clinical History

**Figure 1:**
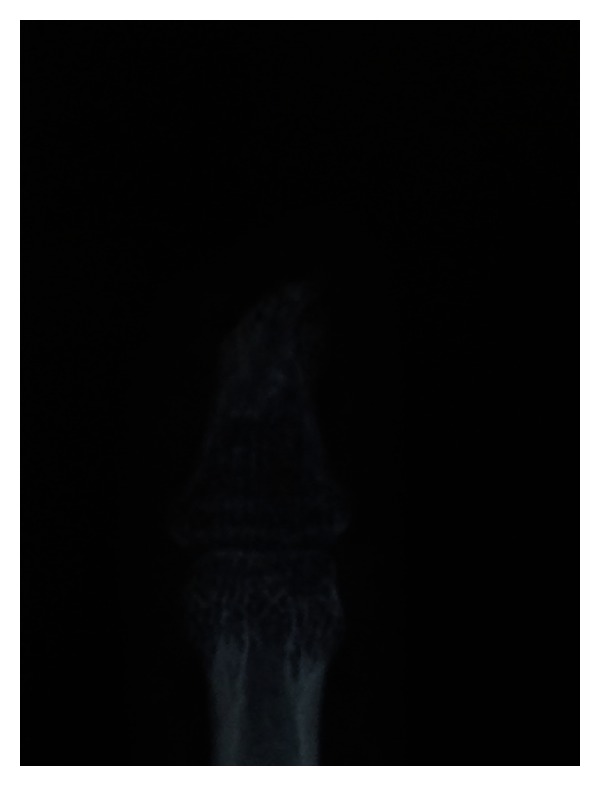
Erosion of the distal phalanx beneath the epithelioid sarcoma.

A 14-year-old girl appeared in the Plastic Surgery Department of Evaggelismos Hospital with a partly subungual, painless, and ulcerated dermal nodule on her left thumb. The patient reported that the lesion was initially observed three years ago and that it was periodically diagnosed and treated as an “infected wart” that failed to heal despite the repeated therapeutic efforts. An X-ray was performed (Figure 1) that showed the characteristic distortion and erosion of at least half of the distal phalanx beneath the dermal nodule. A partial biopsy of the subungual tissue was performed and it was sent to the Pathology Department of Evaggelismos Hospital.

### 2.2. Pathological Findings

**Figure 2:**
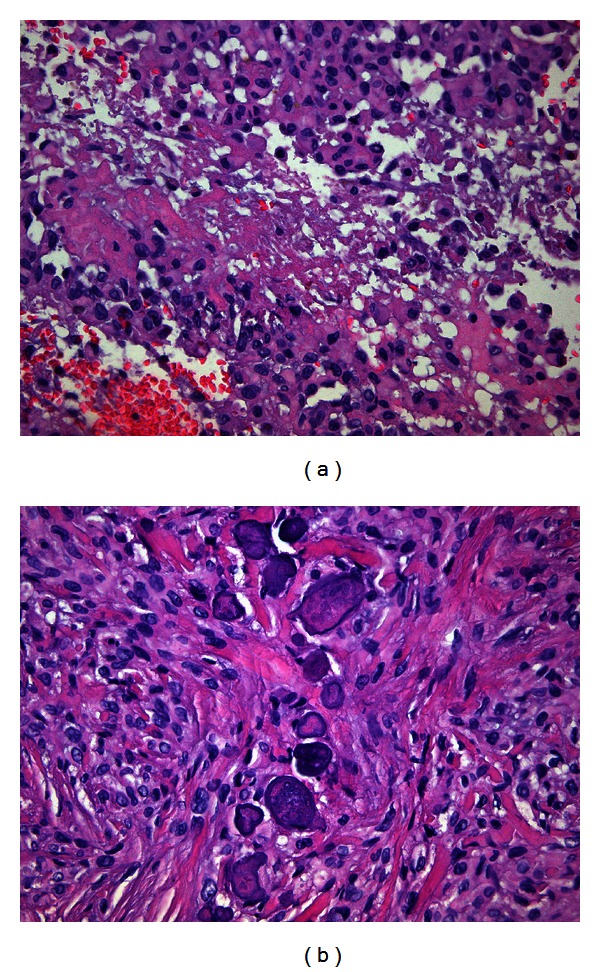
(a) Epithelioid cells with eosinophilic cytoplasm arranged around central necrosis (H&E, ×200). (b) Spindle cells with focal microcalcifications (H&E, ×200).

The neoplasm was located in the dermis underneath the ulcerated squamous epithelium and was composed partly of spindle cells arranged in fascicles and partly of epithelioid, polygonal cells with abundant glassy eosinophilic cytoplasm, an eccentric nucleus with vesicular chromatin and occasional but not prominent nucleoli. Some cells attained “rhabdoid” morphology while others were arranged around central necrosis (Figure 2(a)). Few microcalcifications were observed without osteoid formation (Figure 2(b)). 

### 2.3. Diagnostic Immunohistochemical Markers

The broad immunohistochemical study included the following markers: INI1 (MONOSAN-SANBIO, clone MRQ-27, dilution 1 : 40), Vimentin (DAKO, clone V9, dilution 1 : 4000), cytokeratin CK5/6 (DAKO, clone D5/16B4, dilution 1 : 20), cytokeratin CK8.18 (MONOSAN-SANBIO, clone SD3, dilution 1 : 80), CK19 (DAKO, clone RCK108, dilution 1 : 80), pankeratin AE1/AE3 (DAKO, clone AE1/AE3, dilution 1 : 100), CK7 (DAKO, clone OV-TL12/30, dilution 1 : 80), CK34*β*E12 (DAKO, polyclonal, dilution 1 : 40), CK20 (DAKO, clone K20.8, dilution 1 : 20), CK17 (DAKO, clone E3, DILUTION 1 : 20), EMA (DAKO, clone E29, dilution 1 : 50), Ca-125 (NOVOCASTRA, clone OV185:1, dilution 1 : 50), podoplanin (DAKO, D2-40, dilution 1 : 20), pCEA (DAKO, polyclonal, 1 : 4000), transducin-like enhancer protein 1/TLE1 (ABCAM, polyclonal, 1 : 600), CD99 (DAKO, clone 12E7, dilution 1 : 80), Desmin (DAKO, clone D33, dilution 1 : 60), SMA (DAKO, clone 1A4, clone 1 : 500), Myogenin (SANTA-CRUZ, clone FSD, 1 : 1000), S-100 (DAKO, polyclonal, dilution 1 : 2000), CD34 (DAKO, clone QBEnd10, dilution 1 : 40), CD56 (ZYMED, clone 123C3, dilution 1 : 50), p63 (DAKO, clone 4A4, dilution 1 : 80), bcl-2 (DAKO, clone 124, dilution 1 : 160), Glypican-3 (ZYTOMED, clone 1G12, dilution 1 : 40), CD31 (DAKO, clone JC70A, dilution 1 : 60), GCFDP-15 (NOVOCASTRA, clone 23A3, dilution 1 : 40), Inhibin-a (DAKO, clone R1, dilution 1 : 20), Glut1 (ZYTOMED, clone SPM498, dilution 1 : 200) and ki-67 (DAKO, clone MIB-1, dilution 1 : 100). 

### 2.4. Fluorescent In Situ Hybridization

We used the Vysis break apart probe kit for the detection of the t(X;18) translocation of synovial sarcoma.

### 2.5. Immunohistochemical Findings

**Figure 3:**
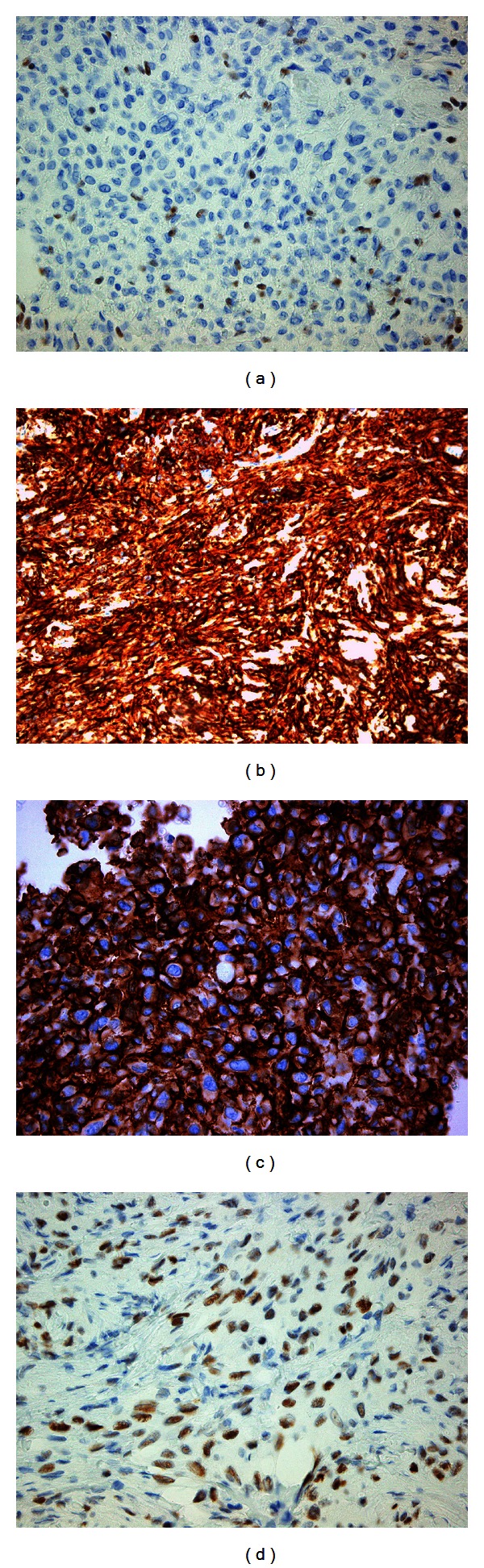
(a) Loss of protein INI1 in neoplastic cells. Endothelial cells retain positivity (IHC, ×400). (b) Ca 125 expression (IHC, ×400). (c) Podoplanin expression (IHC, ×400). (d) TLE-1 expression (IHC, ×400).

All the neoplastic cells exhibited loss of INI1 protein (Figure 3(a)) while they were positive for Ca-125 (Figure 3(b)), Podoplanin (D2-40) (Figure 3(c)), Vimentin, EMA, TLE1 (Figure 3(d)), Glut1, pankeratin AE1/AE3, low molecular weight cytokeratins CK8.18 and CK19 and high molecular weight cytokeratin CK34*β*E12. Several neoplastic cells also exhibited cytoplasmic positivity for high molecular weight cytokeratin CK5/6, pCEA and weak cytoplasmic reactivity for CD99. On the contrary the neoplastic cells were completely negative for CK17, CK7, CK20, S-100, CD34, Desmin, SMA, Myogenin, CD56, p63, bcl-2, Glypican-3, and Inhibin-a. Proliferation index ki-67 was approximately 30%.

### 2.6. Cytogenetic Findings

All of the examined nuclei appeared with one or two fused signals. There was no detection of a split between the telomeric and centromeric end of the SYT gene; thus translocation of the SYT gene was not proven.

### 2.7. Treatment

The patient underwent a surgical excision of the distal phalanx of the thumb that showed, as expected, the infiltration of the underlying bone. CT scan did not reveal any metastasis and the patient is currently placed under follow up.

## 3. Discussion

ES poses a diagnostic challenge for the pathologist especially in small biopsies because of its epithelioid cytologic features that resemble a carcinoma or its central necrosis that can imitate an epithelioid granuloma [1]. The strong reactivity of the neoplastic cells for low and high molecular weight cytokeratins along with the focal pCEA expression can mislead the diagnostic process, especially in CD34 negative cases if additional immunohistochemical markers are not done [5]. The loss of expression of the protein SMARCB1/INI1 characterizes ES but it has never been described in either squamous cell carcinomas or adnexal skin neoplasms. 

Tumors with loss of the SMARCB1/INI1 protein include myoepithelial carcinomas, malignant rhabdoid tumors, atypical teratoid tumors of the central nervous system, renal medullary carcinomas, a subset of malignant peripheral nerve sheath tumors, and extraskeletal myxoid chondrosarcomas, while reduced nuclear expression has also been described in synovial sarcomas [9, 10]. Transducin-like enhancer protein 1 (TLE1) is a protein encoded by the TLE1 gene that appears to be a sensitive but not specific marker for synovial sarcoma. In our case, because of the TLE1, EMA, CD99, and cytokeratin positivity and despite the lack of CD56 and bcl-2 expression which usually manifests in synovial sarcomas, we performed fluorescent in situ hybridization for the detection of the t(X;18) translocation, the absence of which excluded the possibility of the epithelial-type of synovial sarcoma [5]. 

The coexpression of EMA and Vimentin characterizes ES but both markers can also be positive in a variety of adnexal neoplasms that must be taken in consideration in the differential diagnosis of an ulcerated nodule that develops in the dermis. Our case exhibited positivity for Podoplanin (D2-40), a marker of lymphatic endothelium and mesothelial cells that can be expressed in Kaposi's sarcoma, in seminoma, in some cases of epithelioid hemangioendothelioma, and in the epithelioid variant of fibrous histiocytoma [11]. Podoplanin expression has only been rarely reported in epithelioid sarcoma [12], and it must be taken in consideration because its expression along with EMA characterizes neoplasms that derive from the skin adnexa [13, 14]. The negativity of epithelioid sarcoma for p63, which is usually positive in adnexal neoplasms, as well as the loss of the protein SMARCB1/INI1 excludes the possibility of an adnexal neoplasm. 

Additionally our case exhibited cytoplasmic positivity for Ca 125, a relatively nonspecific antigen that is expressed in epithelial ovarian cancer, but it is also raised in endometriosis, inflammatory conditions, and some gastrointestinal carcinomas. Kato et al. studied a series of 78 sarcomas including two cases of rheumatoid nodules all proven to be negative for Ca 125. Rare reports describe THE expression of Ca 125 in isolated cases of rhabdomyosarcoma and desmoplastic small round blue tumor. This evidence appears to be of value for the differential diagnosis of ES from neoplasms that also exhibit loss of INI1 protein [15, 16]. 

Glut1, a glucose transporter protein, was positive in our case and has been reported positive in about 50% of ES but has little value in the differential diagnosis from its dermal mimics as it is also expressed in granuloma annulare and rheumatoid nodules, in benign fibrous histiocytomas and squamous cell carcinomas while it is negative in atypical fibroxanthomas and nodular fasciitis [17]. Finally, the negativity of our case for glypican-3 is a finding consistent with previous reports for this marker in distal ES. On the contrary, glypican-3 has been found positive in a significant percentage of malignant rhabdoid tumors; thus, it seems to be a useful marker, along with Ca 125, for the distinction of ES from malignant rhabdoid tumors [18].
